# Enhancing LED spectral output with perylene dye-based remote phosphor

**DOI:** 10.1038/s41598-023-37956-7

**Published:** 2023-07-05

**Authors:** Jonathan Trisno, Darren C. J. Neo, Maxine M. X. Ong, Ray J. H. Ng, Christina Y. L. Tan, Isabelle S. H. Lee, Hong Son Chu, Ee Jin Teo

**Affiliations:** 1 Institute of High Performance Computing (IHPC), Agency for Science, Technology and Research (A*STAR), 1 Fusionopolis Way, #16-16 Connexis, Singapore, 138632 Republic of Singapore; 2 Institute of Materials Research and Engineering (IMRE), Agency for Science, Technology and Research (A*STAR), 2 Fusionopolis Way, #08-03 Innovis, Singapore, 138634 Republic of Singapore; 3 Arianetech Pte. Ltd, 102E Pasir Panjang Road, #08-02 Citilink, Singapore, 118529 Republic of Singapore

**Keywords:** Materials science, Optics and photonics

## Abstract

LEDs offer a wide range of spectral output with high efficiencies. However, the efficiencies of solid-state LEDs with green and yellow wavelengths are rather low due to the lack of suitable direct bandgap materials. Here, we introduce and develop perylene-enhanced green LEDs that produce a higher wall-plug efficiency of 48% compared to 38% for a solid-state green LED. While the wall-plug efficiency of the perylene-enhanced red LED is still lower than that of a solid-state red LED, we demonstrate that remote phosphor colour converters are effective solutions for targeted spectral tuning across the visible spectrum for horticultural lighting. In this work, we retrofit existing white LEDs and augment photosynthesis via spectral output tuning to achieve a higher red-to-blue ratio. Our results show a significant improvement in plant growth by up to 39%, after a 4-month growth cycle. We observe no visible degradation of the colour converter even under continuous illumination with a current of 400 mA. This opens up new opportunities for using perylene-based colour converters for tuneable illumination with high brightness.

## Introduction

Artificial lighting has progressed from the incandescent light bulb, to fluorescent lamps and to light emitting diodes (LEDs). Mankind has benefited from this technological advancement not just in terms of a boost in energy efficiency, but also a surge in the range of applications^1,2^: mobile gadgets and laptops, projectors, optical communications, and even grow lights for agriculture, just to name a few. However, one of the most impressive features of LEDs is that they offer a wide range of colours. This is achieved by using different semiconductor materials, that have different band gaps, as the active emissive material and thus producing different emission colours. This is different from the concept of applying filters to a broadband source like a fluorescent lamp to get the desired colours, which results in a loss of energy.

Nevertheless, LEDs do have a problem called the “green gap”^3,4^, which is a result of the lack of a suitable direct bandgap material for the emissive layer. Generally, solid-state LEDs in the range of 530–580 nm (i.e. green to yellow) perform poorly, in terms of radiant efficiency, as compared to blue and red LEDs. Blue and red LEDs have efficiencies of more than 50%, while green and yellow LEDs have rather low efficiencies below 40%^4^. One way to overcome the low efficiency of green solid-state LEDs is to apply a phosphor, either an on-chip or a remote colour converter. These techniques are already utilised in many LED products. White LEDs can be produced by using blue LEDs embedded with on-chip phosphors, e.g. yellow Ce:YAG^5,6^. Phosphor-converted white LEDs have been shown to produce natural colours with high colour rendering index (CRI) and high efficiency of 100 lm/W^7^. Nanoco introduced red quantum dots in their white LEDs to produce warm white LEDs with a high CRI^8^. Another example would be the use of green and red quantum dots (QDs) as colour converters for blue organic LEDs (OLEDs) in the latest display tech: QD-OLED^9,10^. Here, we show that by using a certain class of organic dyes—perylene-based dyes^11–13^—we can achieve a better wall-plug efficiency for green LEDs. We choose perylene-based dyes, as they have been shown to exhibit strong light absorption in the UV–visible spectrum, high photoluminescence quantum yield (PLQY), and high photochemical and thermal stability^14–17^. The perylene-based dye is introduced into a polymer matrix host to form a remote phosphor and is placed at a 4 mm distance away from the LED chip. In this configuration where the remote phosphor is mounted close to the LED, the LED is called fluorescence-enhanced LED or F-LED for short, and the remote phosphor is termed fluorescent colour converter (FCC).

In this paper, the F-LED would be investigated for spectral tuning to improve artificial lighting quality and efficiency, and to find out how they could be beneficial in certain applications, such as in agriculture^18^. The market value for LEDs as grow lights for indoor farming, as well as supplemental lighting, has grown tremendously over the years^12^. With more investment coming into this space of controlled environment agriculture, there is an increased awareness of how different light spectra affect crop growth^19^ and indoor farmers would often like to find out what the optimal light intensity and spectrum are for a specific crop. Research studies have thus been conducted on using phosphors and up-conversion particles to tune the light spectra for plants^20–22^ and microalgae^23–25^. These include coating a film of NaYF_4_:Yb,Er up-conversion particles onto leaves^20^ and replacing the roof of a greenhouse with a plastic film with Ca_1−k_Sr_k_S:Cu^+^,Eu^2+^ phosphor additive^22^. Alternatively, applying different coloured LEDs as a multi-wavelength tuneable luminaire requires addressing the different coloured LEDs’ voltages and wattages, which could be cost prohibitive for applications to a large-scale indoor farm. We approach this problem by introducing our FCC as an “on-demand” passive colour tuning. This solution exhibits several advantages: (i) it is low cost, (ii) possesses a great degree of customisation in wavelengths, and (iii) only needs a single type of light source (e.g. blue, UV, or white), removing the need for complex circuitry catering to different turn-on voltages of different colour LEDs. We demonstrate this concept of spectral tuning in a case study where we evaluate the effectiveness of using a red FCC to tune the spectrum of white LEDs for indoor plant growth.

## Results and discussion

### Perylene-based fluorescent colour converter

Perylene-derived dyes are our material of choice for luminescent colour converters because of the following few reasons: (i) It has a high photoluminescence quantum yield (PLQY), (ii) is relatively stable, (iii) is highly soluble in most organic solvents (polar and non-polar) and (iv) has a high working temperature, and hence would be compliant with manufacturing processes like extrusion needed for scaled-up production of the FCCs^26^. In our previous experiments, a variety of dyes was trialed, including coumarin and 4-Dicyanomethylene-2-methyl-6-p-dimethylaminostyryl-4*H*-pyran (DCM) derivative dyes. However, despite their high quantum efficiency and protection in a polymeric matrix, many of these as-purchased commercial dyes suffer from low photochemical stability, which renders them unsuitable for our proposed application with long term high excitation intensity.

**Figure 1:**
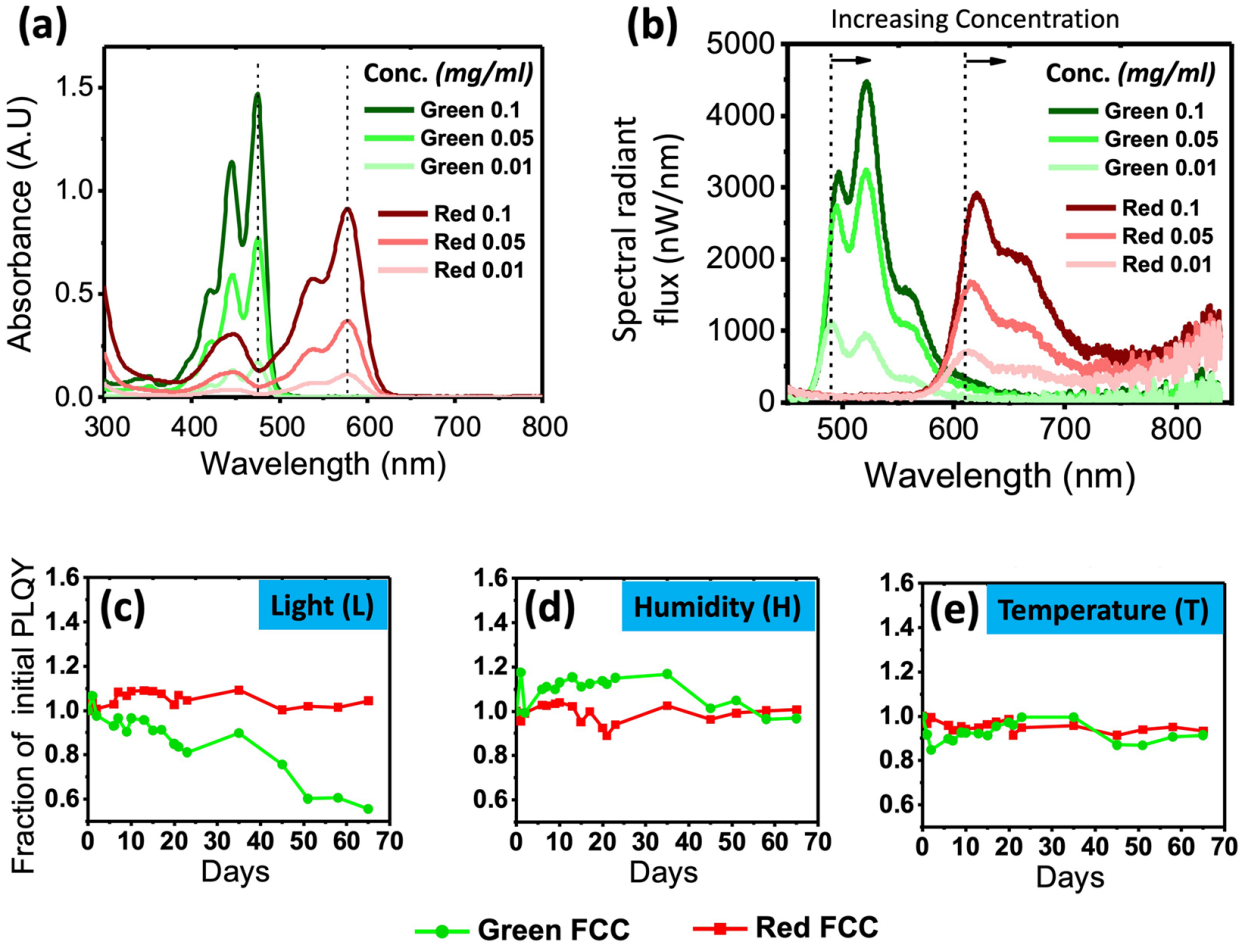
(**a**) Absorption curves of various concentrations of red and green perylene dye in chloroform. (**b**) Corresponding emission curves of the same materials measured in spectral flux (nW/nm) with an excitation wavelength of 405 nm. Dotted lines are added as visual guides and show that there is a negligible shift in absorption peak but a significant red-shift of emission peak as the concentration increases. Stability data of red and green FCC coupons subjected to (**c**) blue light soaking [L], (**d**) high humidity [H], and (**e**) heat [T] conditions as laid out in the “Methods” section.

We first prepare green and red perylene-based dye solutions in chloroform to study their range of absorption and emission (Fig. 1a,b). It can be observed that the green perylene dye absorbs blue light (i.e. in the range of approximately 380–500 nm) better than the red dye. The differences in the absorption spectra (peak wavelengths and intensity) of the perylene dyes can be attributed to the difference in functional groups of the molecules (Fig. S1), which caused them to have different HOMO–LUMO orbitals. Specifically at a wavelength of 480 nm, the green perylene resonates better than the red perylene, allowing it to have stronger absorption. Conversely, at 580 nm, the red perylene absorbs strongly, while the green is not absorbing. We also observed that the green perylene has a lower intrinsic quantum efficiency than the red (84.91–95.71% for 0.001 wt%, Fig. S2), which can be attributed to more non-radiative loss due to the higher absorption at the measurement wavelength of 405 nm (See “Methods”). This non-radiative loss is usually a phonon mediated process, such as localized heating, which increases the rate of degradation (e.g. breaking of bonds or oxidation)^27^.

Figure 1a,b also show that the shape of the absorption profile does not change with the concentration, but the emission red-shifts as the dye concentration increases. This shift can be explained by the overlap between the absorption and emission of the fluorophore, causing re-absorption. The higher the concentration, the higher the probability for re-absorption of emitted (converted) light, and the more non-radiative loss occurs. Therefore, we observed that a higher concentration gives a more red-shifted emission spectrum and a lower PLQY^28^. As the green perylene has a lower intrinsic quantum efficiency than the red, the overall PLQY decreases more with increasing concentration (Fig. S2).

Next, we conducted a study to see if the perylene dyes are suitable to be utilised alongside a commercial solid-state LED as a colour converter. This is done by assessing their stability when subjected to different environmental variables: high light excitation (L), high humidity (H), and moderately high temperature (T). The detailed conditions and characterisation approaches are highlighted in the “Methods” section. We formed coupons of red and green perylene dye in PMMA composites with a fixed dye concentration of 0.01 wt% to the PMMA matrix. Figure 1c–e shows a summary of the results for both colour dyes that were investigated. The PLQY values were normalised to the initial PLQY of the composite. The results revealed that the red perylene dye is very stable: it displayed very minimal or no degradation even after 60 days of conditioning to the different environmental variables. When subjected to blue light soaking (L), humidity testing (H), and heat stress (T), it experienced no significant degradation in PLQY, with measurement variations of up to 10%. The variation could be due to the slightly different placement of the sample on the holder of the integrating sphere across different days. This photochemical stability, as well as the resilience to humidity and temperature exposure, are attributed to the huge resonance stabilisation energy and π-π interactions of the aromatic backbone^29^. The green perylene dye composite, however, was less stable, especially under blue light soaking (L). We observed that the green coupons experience a decrease of 45% from the initial PLQY. One reason could be that although the blue light stress intensity is kept the same for both coloured composite coupons, the green perylene dye has a much higher absorption compared to the red and that could lead to increased photo-oxidation^30^. This higher absorption of blue increases the rate of non-radiative transition process, which introduces localized heating, thereby increasing the rate of degradation (e.g. breaking of bonds or oxidation). Additionally, the green perylene molecule has fewer aromatic rings (5 for green, and 11 for red) in its backbone per molecular weight than the red, which contributes to its lower stability. Structural formulas of both dyes are shown in Fig. S1.

### Fluorescence-enhanced LED

**Figure 2:**
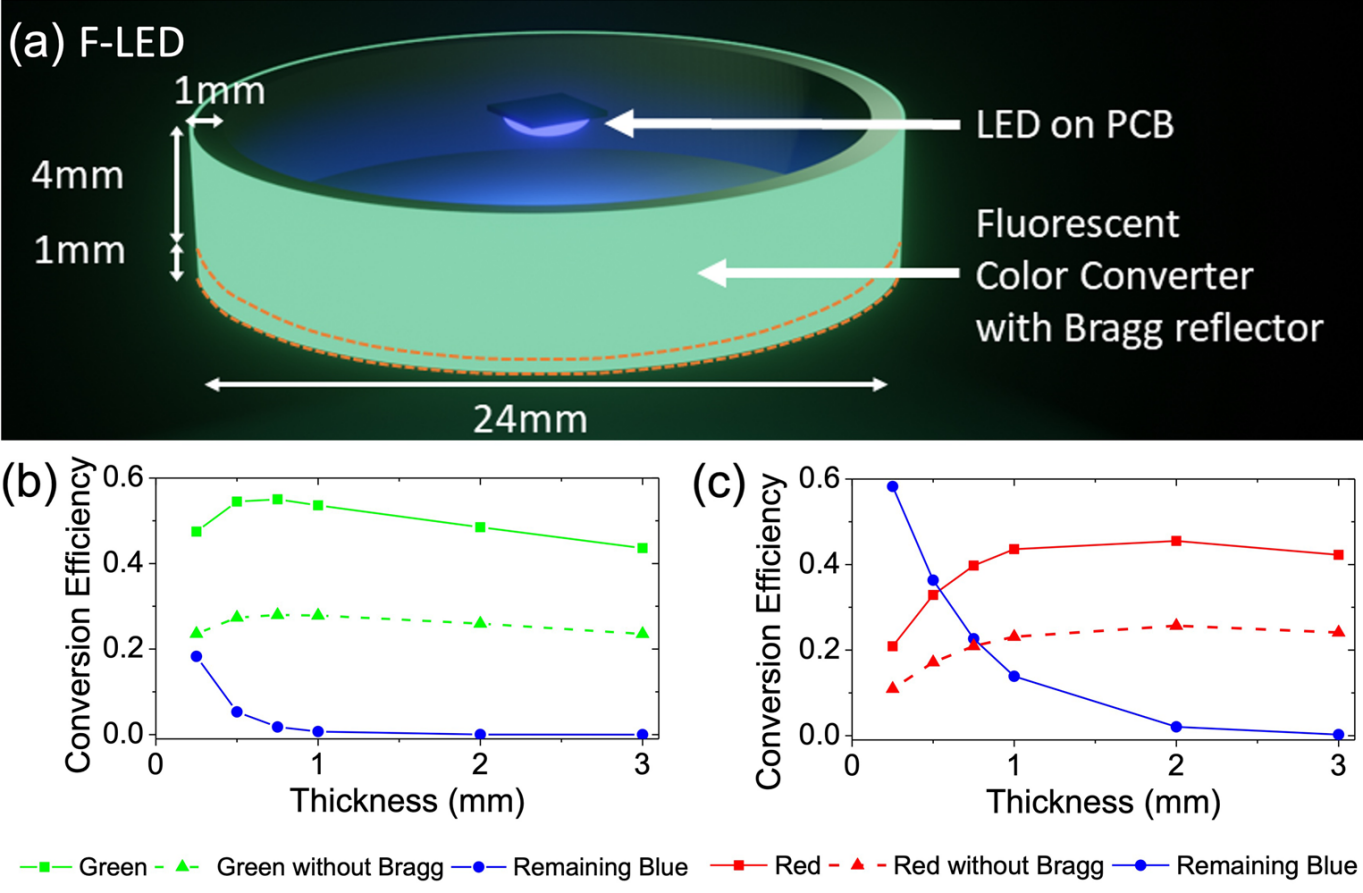
Fluorescence-enhanced LED (F-LED): (**a**) schematic of the F-LED. Simulated conversion efficiency of the FCC as a function of thickness for (**b**) green and (**c**) red colour converters.

Figure 2a shows the simulation setup of the F-LED. We use a blue LED mounted on a printed circuit board (PCB). The LED used is OSRAM GD CS8PM1.14 with its peak wavelength at 451 nm. The FCC component is in the shape of a ‘bottle cap’ with a height of 5 mm and a diameter of 24 mm. The ‘bottle cap’ structure is hollow, leaving an effective colour converter layer thickness of 1 mm, which is 4 mm away from the LED source.

While the fluorescent particles effectively down-convert the blue light into longer wavelengths, they will also scatter the light in all directions. In our simulations, 48% of the down-converted light will be backscattered and this is a significant portion that reduces the efficiency of our desired application. To recover this backscattered light, we incorporate a Bragg reflector layer on the surface of the colour converter that is facing the LED. The Bragg reflector layer acts as a bandpass filter that transmits blue light but reflects green and red lights (Fig. S3). While the Bragg reflector performance is angle-dependent, we expect it to improve the conversion efficiency by up to two times. Here, conversion efficiency (${\eta }_{Conversion}$) is defined as the ratio of the forward-scattered power of the converted light (${P}_{Converted}$) to the incident power (${P}_{Incident}$).

1 $${\eta }_{Conversion}=\frac{{P}_{Converted}}{{P}_{Incident}}$$

This conversion efficiency is mainly dependent on four factors: the extinction coefficient (ε), molar concentration (M), PLQY, and thickness. The molar extinction coefficient describes how well the fluorescent material absorbs light at a particular wavelength per molar concentration. In our study, the extinction coefficients for the green and red dyes for 450 nm excitation wavelength were measured to be 2.43E + 05 M^−1^ cm^−1^ and 1.47E + 05 M^−1^ cm^−1^ respectively, indicating that the green dye absorbs the 450 nm blue light 1.65 times better than the red. Together with the molar concentration values, they define the mean free path of the fluorescent particles, and in turn, define how much of the incident light would be absorbed. The absorption follows the Beer-Lambert law which governs the attenuation of light passing through a material^31^. The photoluminescence quantum yield (PLQY) is defined as the ratio of the number of photons emitted to the number of photons absorbed. The higher the PLQY, the more efficient the down-conversion process is. As previously discussed, the green dye is less efficient than the red, with a PLQY of 85% for green versus 95% for red.

Figure 2b,c show the impact of thickness on the conversion efficiency. The higher conversion efficiency for the green FCC can be attributed to the higher absorption of blue light, resulting in a higher production of down-converted light compared to the red FCC, despite the lower PLQY. The highest conversion efficiency for the green FCC is 55% compared to 46% for the red FCC. This result is based on simulations of both the green and red FCC with the same fluorescent particles concentration of 0.05 mg/mL. However, we should take note that the same weighted concentration (mg/ml) corresponds to different molar concentration (M) values of 9.95E–5 mol/L for green FCC and 4.63E–5 mol/L for red FCC. The difference is due to the green dye having a lighter molar mass of 502.6 g/mol compared to 1079.26 g/mol for red^32,33^. Hence, it is expected for the green dye to have stronger absorption than the red dye because of its higher extinction coefficient and molar concentration.

The effect of thickness on the green FCC is shown in Fig. 2b. We simulated FCCs with 6 different thicknesses (0.25, 0.5, 0.75, 1, 2, and 3 mm). The highest conversion efficiency is 55% for a thickness of 0.75 mm. The continuous and dotted green lines show the comparison between FCCs with and without a Bragg reflector. On average, the Bragg reflector improves the conversion efficiency by 1.9 times. While the green FCC shows very strong absorption, not all of the blue incident light will be absorbed and down-converted. The blue curve shows the ratio of the remaining blue light that is not down-converted to the incident blue light. With increasing thickness, this value will drop, and eventually, all incident light will be down-converted for thickness > 1 mm. This region can be regarded as the saturation region. For thicknesses < 0.75 mm, where a significant percentage of blue light is not fully absorbed, the increasing thickness will contribute to the increasing conversion efficiency of the green FCC (green curves). However, when most of the blue light has been absorbed and down-converted, the conversion efficiency starts to drop linearly with the increasing thickness. This can be mainly attributed to the occurrence of re-absorption, due to the overlap of the absorption spectrum and the emission spectrum at 450–500 nm (Fig. 1a,b). When the thickness increases, the chance of re-absorption increases, and due to quantum losses, the conversion efficiency decreases.

Figure 2c shows the effect of thickness on the red FCC. In this case, incorporating the Bragg reflector also improves the conversion efficiency significantly. For the red FCC, the saturation region where the blue incident light is fully absorbed and down-converted is achieved only for thicknesses > 2 mm. The occurrence of saturation at a larger thickness is expected as the red FCC has a lower extinction coefficient and lower molar concentration than the green FCC. When the thickness is increased in the non-saturated region, the conversion efficiency increases from 21% at 0.25 mm to 46% at 2 mm. When we further increase the thickness to 3 mm, the conversion efficiency decreases, but at a slower decay rate than for the green FCC. While the red FCC also experiences re-absorption due to overlap between the absorption and emission spectra from 580 to 620 nm, the effect is less significant due to the higher quantum efficiency (PLQY) of 95% for the red FCC compared to 85% for the green.

**Figure 3:**
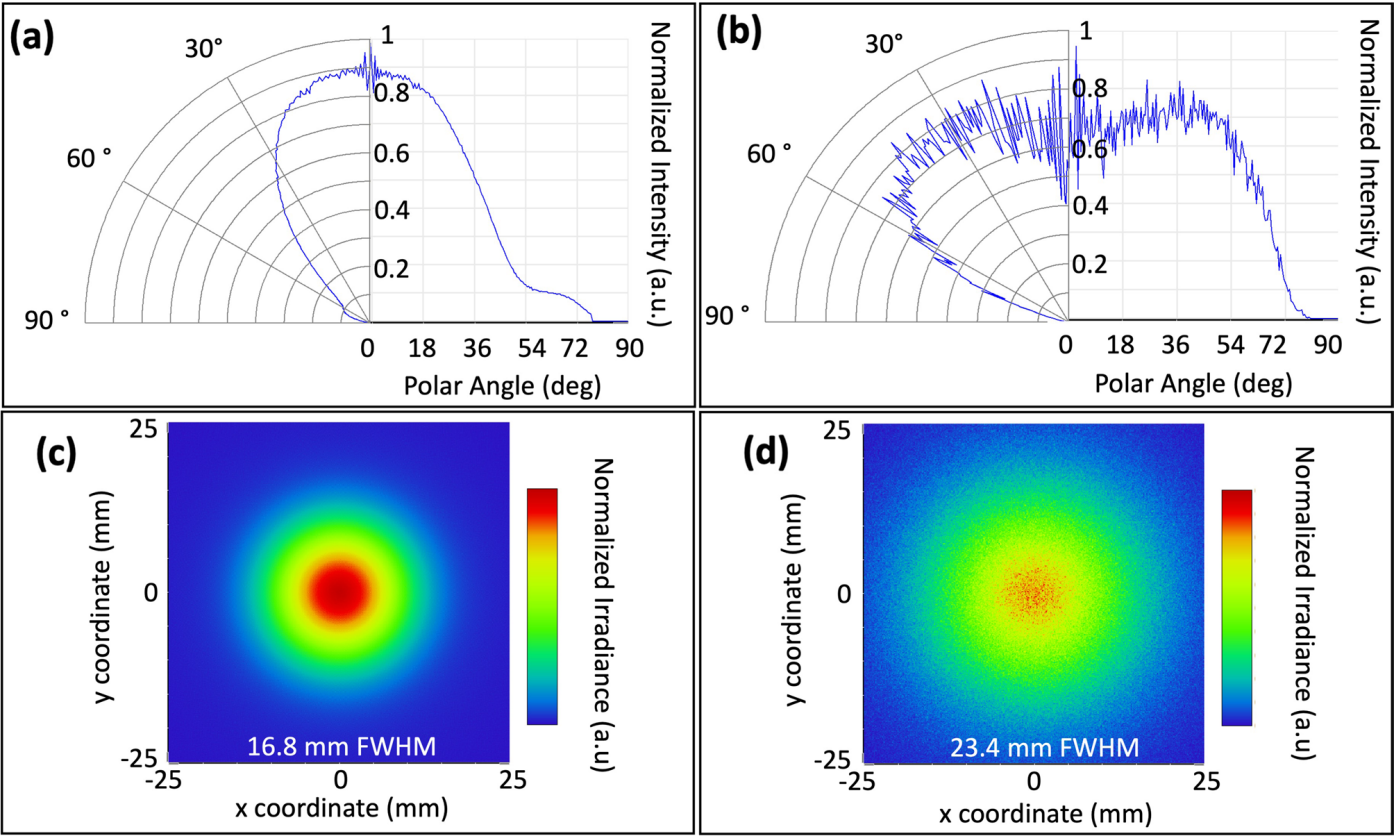
Simulated radiation characteristics and irradiation patterns of (**a**,**c**) blue LED and (**b**,**d**) F-LED (blue LED + green FCC).

The colour converter will change the radiation characteristic of the LED. Figure 3a shows the radiation characteristic of the blue LED with a half-angle divergence of 40° calculated at half maximum. With the green FCC layer, the F-LED shows a 65° half-angle divergence (Fig. 3b). The F-LED shows a flatter profile from 0° up to 54°, indicating a more uniform illumination. Figure 3c,d show the irradiance measured at a 15 mm distance from the source, for blue LED and F-LED (blue LED + green FCC) respectively. The figures show a more uniform illumination for F-LED with an FWHM of 23.4 mm compared to 16.8 mm for the blue LED. For applications in farms, the more diffused and uniform illumination could be beneficial, as this will reduce hotspots and improve plant growth.

**Figure 4:**
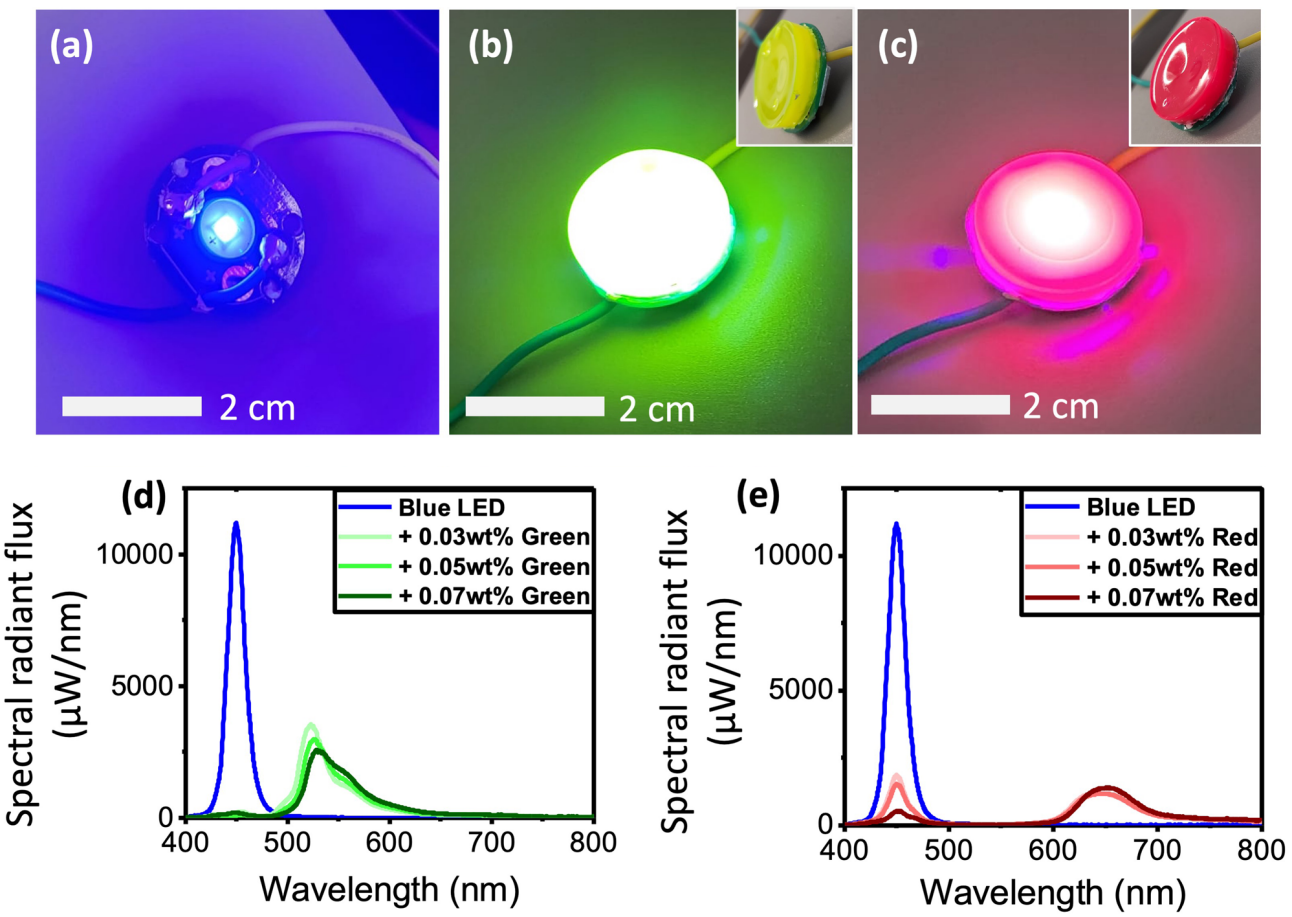
(**a**) Solid-state blue LED, converted to (**b**) green F-LED and (**c**) red F-LED using FCCs (with fixed camera setting), with inset showing the bottle cap structure of the FCC. Comparison of the spectral responses of solid-state blue LEDs with (**d**) green and (**e**) red F-LEDs with varying FCC dye concentrations.

By fabricating cap-like FCC pieces with perylene dye embedded into PMMA, affixing a Bragg reflecting layer on the inner side of the cap (similar to the simulation set-up), and then putting them over an OSRAM GD CS8PM1.14 (blue) LED, we tested the optical as well as the electrical response of F-LED. Figure 4a shows the solid-state blue LED, which changes in colour when fitted with green or red FCCs, to become an F-LED (Fig. 4b,c). The F-LEDs and solid-state LEDs are tested by collecting all emissions via an integrating sphere and measuring them with a calibrated spectrometer. From this, we can figure out the radiant energy emitted from the device. With a sourcemeter to supply electrical power to the devices, we can track the electrical power consumption. Ultimately, we can calculate the wall-plug efficiency based on Eq. (2):

2 $${n}_{wall plug}=\frac{{P}_{light\, emitted}}{{P}_{electricity\, consumed}}=\frac{{P}_{light\, emitted}}{I\cdot V}$$

Table 1 shows the measurement results of F-LEDs compared to solid state LEDs. We can see that for every F-LED as compared to the base solid-state LED (blue), there is a drop in wall-plug efficiency due to non-ideal PLQY and the loss of some light that is not captured by the integrating sphere. However, in the case of green F-LED, we show that it could be more efficient compared to the solid-state green LED. The same cannot be said of the red F-LED because the red and blue solid-state LEDs are very efficient themselves. When comparing the green to the red F-LED, we observe that the green F-LED is brighter than the red (Fig. 4b,c). Measurement results in Fig. 4d,e also show that the green F-LED produces a brighter spectrum than the red, with higher peaks. We observe that the conversion of blue photons to green is very high as there is almost no blue peak present. This is important because if the absorption of blue is weak, a thicker or higher concentration FCC layer would be needed, and this would compromise the PLQY of the FCC. Thus, resulting in a considerable drop in wall-plug efficiency. Conversely, in the case of the red FCC (Fig. 4e), owing to its weaker absorption in the blue wavelengths, the conversion is not complete at similar concentrations. The increase in conversion efficiency with concentration for the red FCC demonstrated in Table 1 is indicative that the FCC is not in the saturated region yet, *i.e*. the blue has not been fully absorbed. The wall-plug efficiency of the red F-LED is lower compared to the solid-state red LED.

**Table 1:**
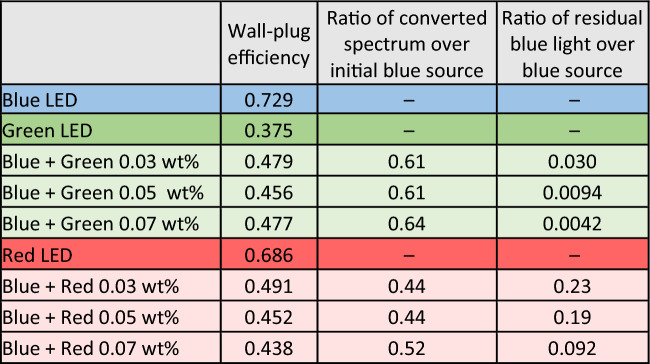
Wall-plug efficiencies of solid-state LEDs, compared with green and red F-LEDs.

### Farm trial: enhancing plant growth with F-LED

**Figure 5:**
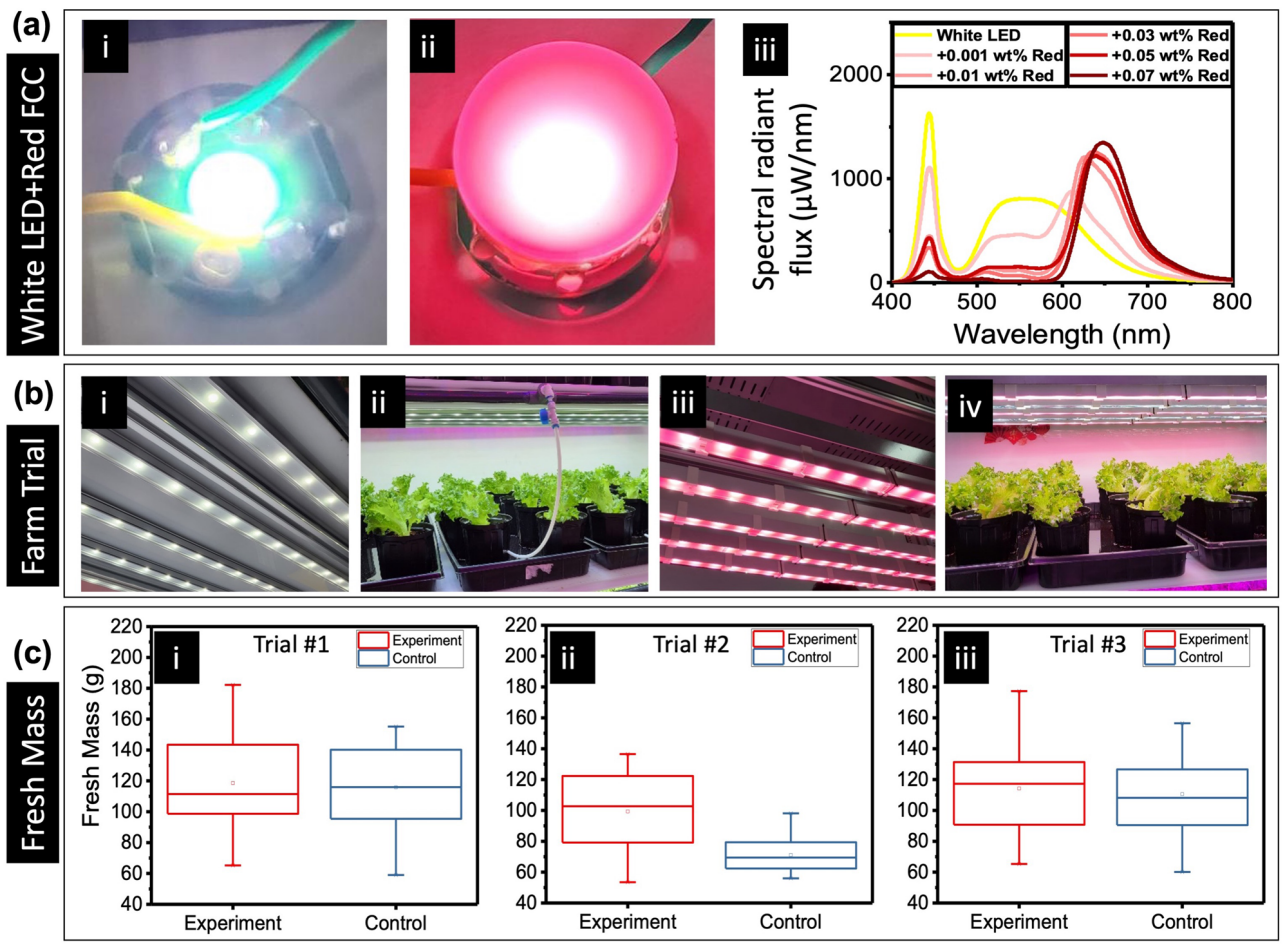
(**a**) White LED + red FCC: [i] White solid-state LED, [ii] spectral tuning of white LED with red FCC, [iii] spectral responses of white LEDs with red FCCs with varying concentrations. (**b**) Farm trial: [i] 4700 K white LED light strip (for control), [ii] lettuce under control conditions, [iii] 4700 K white LED light strip with red FCCs (for experiment), [iv] lettuce under experimental conditions. (**c**) Fresh mass obtained over 3 independent indoor farm trials: [i] trial #1, [ii] trial #2, [iii] trial #3.

Consequently, we found a niche application for the red FCC. White light LEDs (especially cool white LEDs) only have a small amount of the red component compared to blue. Since white light sources have a huge number of photons at the green and yellow wavelengths, when they are utilised as grow lights for indoor farming, these green and yellow photons are greatly wasted as green plants do not absorb them (mostly reflected) as much as in the blue and red^34,35^. Looking at the absorption spectrum of red perylene, we see that its maximum absorption lies in the 500–600 nm range. Therefore, by applying red perylene as an FCC for white LEDs, we can convert the underutilised yellow and green wavelengths into red without reducing much of the total number of photons reaching the crops (Fig. 5ai,ii). The lower wall-plug efficiency is also due to the fact that we cannot apply a Bragg reflector film to enhance light extraction in this case as it would block of the transmission of green and red photons from the white LED itself. Simply put, the total amount of useful photons for plant growth increased. This is despite ~ 20% lower wall-plug efficiency of the white LED + red FCC compared to just the white LED on itself (Table S1). Our experiment does show a significant change and a visible red peak in the output spectrum of the converted white LED (Fig. 5aiii). As the concentration of red perylene dye increases, conversion increases but again, the PLQY would drop. From this information, we choose the lowest concentration of red perylene dye investigated in our experiment to be used as the FCC for white LED in a farm trial.

With 4700 K white light LEDs as the control lighting setup, the experimental lighting setup is made using strips of 0.001 wt% red perylene placed on the same white LED light bars. To ensure a fair experiment, the same driver power was administered to both setups and the experimental lighting height was adjusted so that both setups achieve the same photosynthetic photon flux density (PPFD) level of 200 µmol m^−2^ s^−1^ as measured from the pot level. The initial and final measurements of the red FCC enhanced white light show a red to blue photon ratio of about 2.7 while the control white LED has a ratio of 1.2. A lettuce variety *“Cristabel”* was chosen for its compactness (both horizontally and vertically) so that the crops would not overgrow and start shading each other. Figure 5bi–iv shows our indoor farm trial setup and Fig. 5c is a summary of the results obtained from the three independent trials. These three trials were conducted consecutively, by comparing the experimental with the control groups, each consisting of 32 plants (see “Methods”). The trials are conducted using the same LEDs and F-LEDs. From the results, we can conclude that because of the improvement in the quality of the spectrum afforded by red FCC enhancement, the fresh mass collected is higher. Based on the mean value, we observe that the FCC-enhanced white light produces between 5 and 39% higher fresh-mass. The variations in the fresh mass are due to slight variations in several possible factors, *e.g.* CO2 content, temperature, water content, and nutrients content, as the trials were conducted consecutively with the same set of FCCs. FCC-enhanced white light has a higher red to blue photon ratio as compared to unconverted white light. Previous studies corroborate our results as they have shown that increasing red light for the growth of leafy greens helps to increase fresh mass and secondary metabolites^36,37^.

At the end of the four-month-long investigation, the red FCCs were returned to the lab to be examined. There were no visible hotspots and the PLQY did not display any significant degradation (Fig. S5). The power consumption was measured to be 100 W and the LEDs are driven by a current of close to 0.43 A with a voltage drop of 230 V. This current level is very much higher than that reported by previous attempts in using perylene dyes on LEDs^38^. These observations from the farm trials show the remarkable stability of the formulated red FCC. While we only demonstrated the effectiveness of retrofitting existing white LED with red FCCs, green FCCs could be beneficial in some applications of agriculture, which is crop dependent: e.g. penetration into deeper layers of leaves to increase photosynthesis, and promoting stem elongation. Hence, our organic phosphor-enhanced LEDs could be a good solution for tailoring to such various scenarios.

To further evaluate the performance of our F-LED in our indoor farm trial, we compare its results with other studies involving light wavelength conversion with phosphors and nanoparticles. Two studies separately using NaYF_4_:Yb,Er up-conversion nanoparticle^20^ and NaYF_4_:Yb,Tm up-conversion nanoparticles^21^ showed an improvement in the photosynthetic rate by 150% (mung bean) and 12% (Arabidopsis thaliana) respectively, while another study on Ca_1−k_Sr_k_S:Cu^+^,Eu^2+^ phosphor showed an increase in yield of 24% (cabbage)^22^. These approaches have significant disadvantages compared to our F-LED design, which integrates the phosphor particles with the LED source in a compact manner. The first approach of coating the leaves with a film of nanoparticles requires spraying the particle solution directly onto the leaf surface, which is inconsistent and slow. The up-conversion particles used contain yttrium and ytterbium, which have potential toxicity to biological organisms^39,40^. All the above studies were also based on a solar light source, which may not be suitable for intensive indoor farming environments. In addition, the durability of the phosphors and nanoparticles was also not investigated, whereas the perylene red and green dyes have been shown in our study to have good durability for up to 40 days in high heat, humidity, and light conditions.

## Conclusion

In conclusion, we have demonstrated the viability of using perylene dyes as remote phosphors to alter the LED output spectrum. In particular, we have showed that the green F-LED could be even more efficient than its solid-state counterpart. Our study provides an alternative to commercial inorganic on-chip phosphor, by developing a low-cost, organic-based phosphor-enhanced illumination. Instead of fixing the phosphor onto the LED die, we utilised a layer of organic phosphor-embedded polymer at a distance away from the source, which enables retrofitting to existing LEDs. This approach allows easy-changing of phosphor to tune the spectrum that has been optimised for specific applications, e.g. various species of crops. We demonstrated organic compounds-based colour converters can be efficient, durable, and cost-efficient for applications in spectral tuning. However, the long-term stability of the green FCC is to be addressed perhaps by studying the degradation mechanism in detail and introducing additives in the polymer matrix to strengthen durability. With even higher stability, we might even be able to put the FCC on-chip, reducing the need for components like the Bragg reflector layer. The benefits of F-LED can also be seen in using the red perylene FCC to alter the spectrum of white light LEDs, cutting down on green photons while giving a boost in red photons, which is better absorbed by the plants. Our results showed, on average, an 18% increase in yield when lettuce is grown under a red converted white LED as compared to a controlled white LED with the same power. Produced in a low-cost manner, these FCCs can be an ideal component in LED fixtures to tune the spectrum of output light efficiently.

## Methods

### Materials

Organic dyes, fluorescent red #94720 and fluorescent yellow #94700 were purchased from Kremer Pigmente (used as received). Poly(methyl methacrylate) (PMMA) pellets were purchased from Chi Mei Corporation, Taiwan.

LED chips were purchased from OSRAM and were mounted on a starboard. Red (OSRAM GH CSSPM1.24) OSLON^®^ SSL 120, green (OSRAM GT CSSPM1.13) OSLON^®^ SSL 120, blue (OSRAM GD CSSPM1.14) OSLON^®^ SSL 120, and white 5700 K (GW CS8PM1.PM) OSLON^®^ were used.

The colour converters were made in upscaled batches via a solution processing method, with powdered PMMA (of selected molecular weight) and selected dyes (in desired weight percentage) dissolved in an organic solvent (chloroform). The coupons were then cast and molded into the desired geometry, and allowed to slowly dry in an aluminium tray.

The photoluminescence spectrum and photoluminescence quantum yield (PLQY) were measured with an integrating sphere connected to a calibrated spectrometer (Ocean Optics Usb4000), with the samples probed using a 405 nm LED with a power output of 24.3 mW. The same setup was used to measure the efficiency of LEDs and F-LEDs, with the integrating sphere capturing the light emitted from the device while the device was driven by a source meter. The current and voltage (electrical power) were read off from the source meter while the radiant power was taken from the readings on the calibrated spectrometer.

The stability test of the dye-PMMA composites was conducted in 3 independent trials: (i) on a 100 mW cm^−2^ blue light LED table, (ii) in an 85% relative humidity (RH) chamber, (iii) in a 65 °C oven. At specific intervals, coupons of test composites that were placed in the as-stated conditions were removed momentarily and a PLQY measurement was taken before putting them back to their test conditions.

Lettuce (variety: *Cristabel*) was grown from seeds to the seedling stage in smaller trays under a 4700 K white light for two weeks, after which healthy seedlings of similar sizes were transplanted into individual soil-based pots to be used for experiments. The seedlings were split between a control group with 4700 K white light LEDs and an experimental group that had 4700 K white light LEDs with red FCCs attached 2 cm above the LED chips. Each group consisted of 32 plants. All plants received the same amount of nutrients and the light source was adjusted in height such that both test and control plants had the same PPFD and daily light integral. The light source power was kept the same at 100 W.

### Simulations

The F-LED design was optimized and tested via ray tracing simulations using the commercial software Zemax OpticStudio Premium v21.2. The design was simulated in Non-Sequential Mode, with a Radial source modelled after the OSRAM GD CS8PM1.14 blue LED. The colour converter layer was placed at a 4 mm distance from the source with a thickness of 1 mm. The colour converter layer is PMMA with an index of refraction of 1.49. The surface facing the source is coated with an angle-dependent Bragg reflector, with data extracted from measurement (Fig. S4). The perylene-derived dyes were modelled based on experimental absorption and emission spectra. The green dye has an 85% quantum yield, a calculated extinction coefficient of 2.58E + 5 M^−1^ cm^-1^ (for 450 nm extinction wavelength), and a phosphor density of 5.99E + 16 cm^−3^, which translates into a mean free path of 0.17 mm at the extinction wavelength. The red dye has 95% quantum yield, calculated extinction coefficient of 1.47E + 5 M^-1^ cm^−1^ (for 450 nm extinction wavelength), phosphor density of 2.79E + 16 cm^-3^, which translates into a mean free path of 0.64 mm at the extinction wavelength. For both dyes, Mie scattering from the scattering particle was considered with a particle index of 1.42, a radius of 2.5 um, and a density of 3.82E + 8 cm^−3^. The simulation was set to consider polarization, ray splitting, and ray scattering. The flux and irradiance pattern were recorded from a 50 × 50 mm monitor placed at 5 and 10 mm distance, respectively, from the FCC.

## Research involving plants

The use of plant parts in the study complies with international, national, and/or institutional guidelines. The only requirement we had from Singapore Government—National Parks Board was obtaining Phytosanitary certification when the seeds were imported into Singapore.

## Supplementary Information

Supplementary Information.

## Data Availability

The data that support the figures and other findings of this study are available from the corresponding authors upon reasonable request.
