# Supplementary material for: Enhancing LED spectral output with perylene dye-based remote phosphor
Source: Sci Rep. 2023 Jul 5;13:10841. doi: 10.1038/s41598-023-37956-7 (PMC10322934; doi:10.1038/s41598-023-37956-7)
Supplement: Supplementary file 1 — Supplementary Information. [file 41598_2023_37956_MOESM1_ESM.pdf]

Supplementary Information

Enhancing LED spectral output with Perylene dye based remote phosphor

Jonathan Trisno[1, *], Darren C. J. Neo[2, *], Maxine M. X. Ong[2], Ray J. H. Ng[1], Christina Y. L. Tan[2], Isabelle S. H. Lee[3], Hong-Son Chu[1, ‡], Ee-Jin Teo[2, †]

[1]Institute of High Performance Computing (IHPC), Agency for Science, Technology and Research (A*STAR), 1 Fusionopolis Way, #16-16 Connexis, Singapore 138632, Republic of Singapore

[2]Institute of Materials Research and Engineering (IMRE), Agency for Science, Technology and Research (A*STAR), 2 Fusionopolis Way, #08-03 Innovis, Singapore 138634, Republic of Singapore

[3]Arianetech Pte. Ltd, 102E Pasir Panjang Road, #08-02 Citilink, Singapore 118529, Republic of Singapore

* Contributed equally

‡ E-mail: chuhs@ihpc.a-star.edu.sg

† E-mail: teoej@imre.a-star.edu.sg

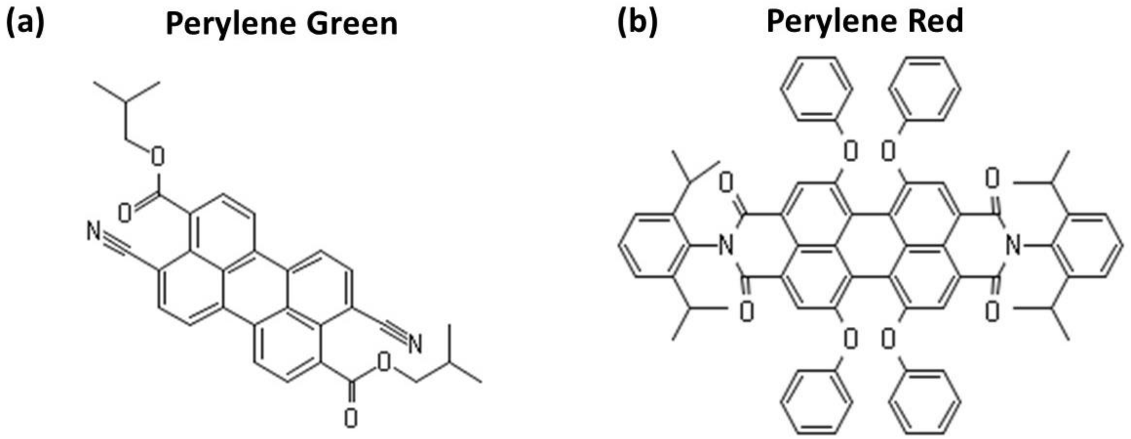

Figure S1. Structural formula of (a) Perylene Green (3,9-bis(2-methylpropyl) 4,10-dicyanoperylene-3,9-dicarboxylate; $C_{32}H_{26}N_2O_4$) and (b) Perylene Red (7,18-bis[2,6-bis(propan-2-yl)phenyl]-11,14,22,26-tetraphenoxy-7,18-diazaheptacyclo[14.6.2.22,5.03,12.04,9.013,23.020,24]hexacosa-1(22),2(26),3,5(25),9,11,13,15,20,23-decaene-6,8,17,19-tetrone; $C_{72}H_{58}N_2O_8$).[1,2]

(a)

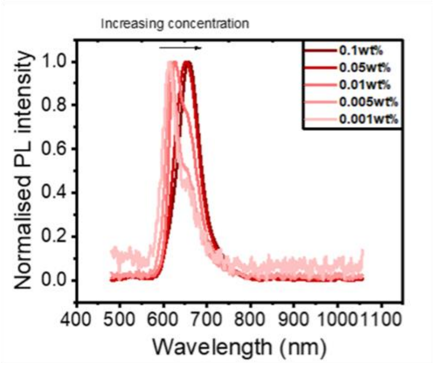

(b)

Sample	PLQY
0.1wt%	84.89%
0.05wt%	89.70%
0.01wt%	90.31%
0.005wt%	93.83%
0.001wt%	95.71%

(c)

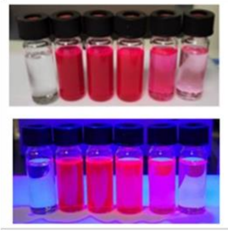

(d)

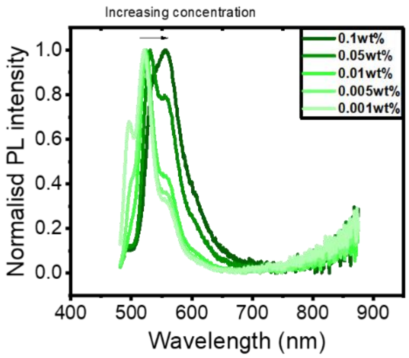

(e)

Sample	PLQY
0.1wt%	54.58%
0.05wt%	59.22%
0.01wt%	71.91%
0.005wt%	73.61%
0.001wt%	84.91%

(f)

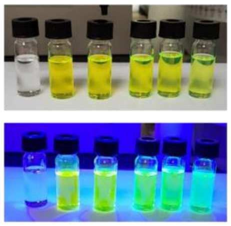

Figure S2. (a) Normalised emission, (b) PLQY and (c) images of red perylene dye in chloroform, with increasing concentration. (d) Normalised emission, (e) PLQY and (f) images of green perylene dye in chloroform, with increasing concentration. The perylene solutions are under white light illumination (top image) and blue light illumination (bottom image) respectively.

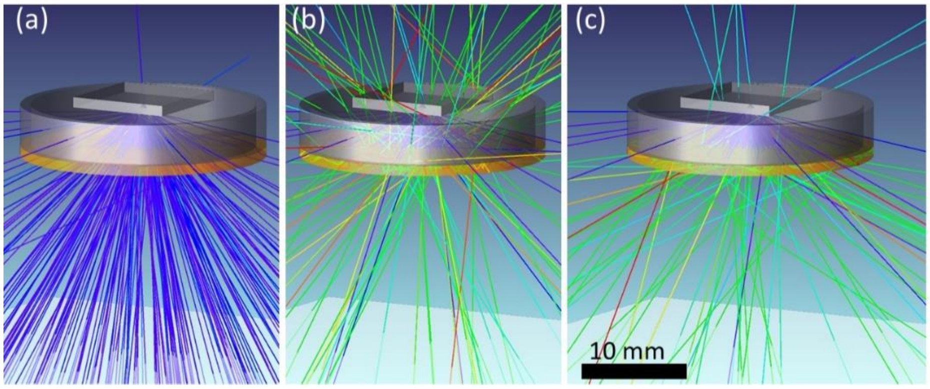

Figure S3. Bragg stack functioning as a band-pass filter to improve directionality. Schematics of light rays passing through (a) pure PMMA resin, (b) colour converter with significant backscattering, (c) colour converter with Bragg coating improving forward-scattering.

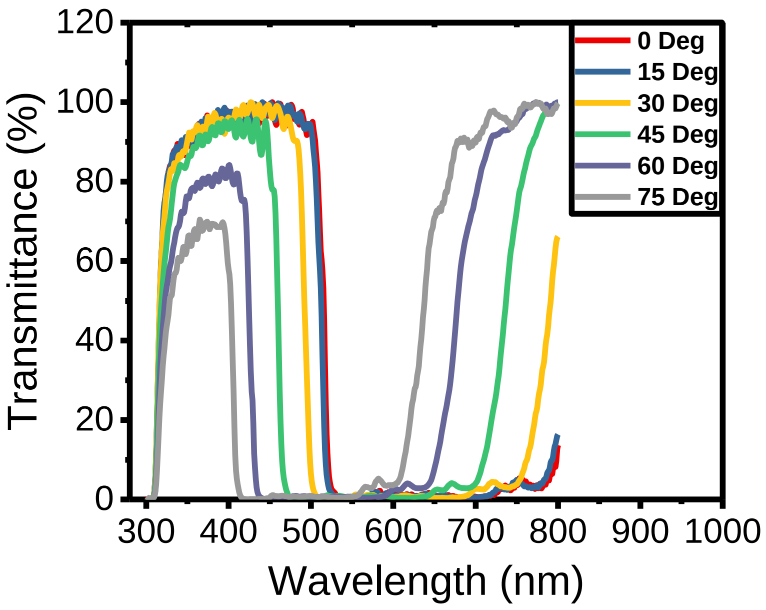

Figure S4. Angle dependent transmittance measurement of the Bragg reflector film utillised in the wall plug measurement experiment. Note that 0 degrees refers to normal incidence of light on the Bragg reflector film. The angles are measured with respect to the normal of the film surface.

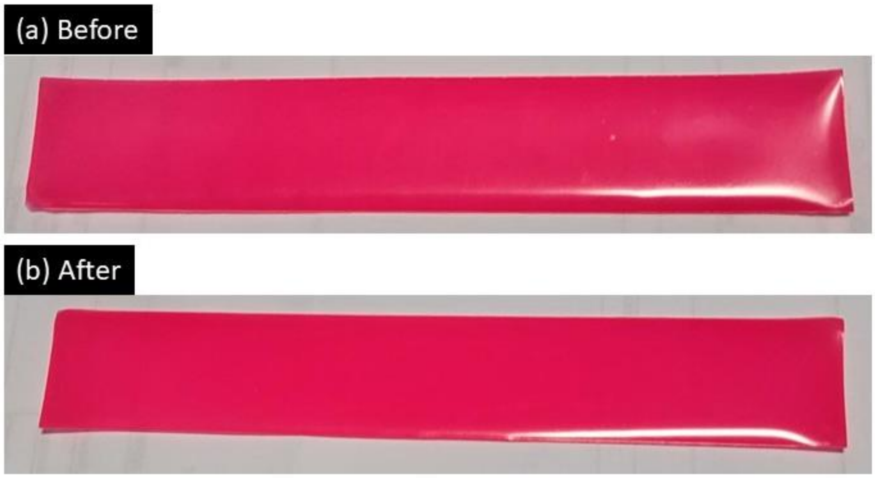

Figure S5. Red FCC in a form of a stripe used in farm trial: (a) before, and (b) after being used in three trials conducted over a 4-month period without any downtime.

Table S1. Wall plug efficiencies of white LED and white LEDs combined with red FCCs.

	Wall plug eff
White LED (4700K)	0.534
White + Red 0.001 wt%	0.487
White + Red 0.01 wt%	0.431
White + Red 0.03 wt%	0.424
White + Red 0.05 wt%	0.453
White + Red 0.07 wt%	0.395

Table S2. Comparison between this work and other spectral tuning solutions

	Perylene-enhanced LED	vs commercial LED with Phosphors	vs Solid-State LED
Phosphor material	Organic dyes	Inorganic materials, rare-earth doped YAG, quantum dots, lanthanides	NA
Green efficiency	48%	>40%	<40%
Spectral tuning	Targeted spectral tuning of white LEDs to produce higher red-to-blue ratio for enhanced crop growth. Mixing of dyes can produce new spectral emission.	Tuning is fixed once LED and phosphor are manufactured.	Requires LED color mixing. Expensive, potentially lower wall-plug efficiency.
Solubility	Readily soluble to form uniform films	Micron and nano sized particles are not as soluble and suffer from aggregation.	NA
Cost	Cost-effective, low-cost dye, cheap to fabricate, can be retrofitted to existing illumination.	More expensive, requires multistep or high energy processes to produce phosphors	More expensive, especially if a tunable spectrum is required.
Other Advantages	Organic compounds can have higher flexibility, temperature resistance, and lower toxicity.	Highly integrated solution allows for streamlined manufacturing and compactness.	More stable

Table S3. Comparison with other works for the green phosphors[3–7]

	Perylene-enhanced LED	Inorganic Materials			Solid-State LED [6]
		Rare-earth doped YAG[3]	Quantum Dots [4]	Lanthanides [5]	
**Material**	Perylene Green	YAGG:Ce-YAG Ceramic	InP	Dy3+/Tb3+ doped	InGaN
**Green wall-plug efficiency**	48%	NA	30%	NA	NA
**External Quantum Eff. (EQE)**	56.5% (calculated)	68.3%	36% (calculated)	32%	24.7%
**PLQY**	85%	97%[7]	96%	NA	NA
**Absorption wavelength**	380-500 nm	345 nm and 440 nm	400-600 nm	452 nm	NA
**PL wavelength**	480-610 nm (with 450 nm excitation)	480-680 nm (with 450 nm excitation)	541 nm, with FWHM 47nm	545 nm and 575 nm (excitation at 452 nm)	559 nm
**Stability study**	65 days of exposure to: (a)Photo: Retain 60% performance (b) Humidity (85%): No degradation (c) Thermal (65°C Oven): No degradation	Thermal stability study: retaining 90% of performance at 200°C	Thermal: Retain 85% of performance at 65°C	NA	NA

Extinction coefficient calculation:

The extinction coefficient is calculated based on the Beer-Lambert law:

$$\varepsilon = \frac{A}{cL}$$

Here, ε is the molar extinction coefficient. It is calculated based on the experimentally measured absorbance A, path length L, and the molar concentration of the sample c, which is in turn calculated based on the equation below:

$$c = \frac{\rho}{M}$$

where the molar concentration c (mol/L) is a function of the mass concentration ρ (g/L) and molar mass M (g/mol).

References:

1. Japan Science and Technology Agency. Lumogen Yellow 083. *https://jglobal.jst.go.jp/en/detail?JGLOBAL_ID=200907049913959905*.

2. Japan Science and Technology Agency. KF-856. *https://jglobal.jst.go.jp/en/detail?JGLOBAL_ID=200907021852192981&rel=1#%7B%22category%22%3A%220%22%2C%22keyword%22%3A%22lumogen%20red%22%7D*.

3. Zhu, Q.-Q. *et al.* YAGG:Ce Phosphor-in-YAG Ceramic: An Efficient Green Color Converter Suitable for High-Power Blue Laser Lighting. *ACS Appl Electron Mater* **2**, 2644–2650 (2020).

4. Choi, Y., Choi, C., Bae, J., Park, J. & Shin, K. Synthesis of gallium phosphide quantum dots with high photoluminescence quantum yield and their application as color converters for LEDs. *Journal of Industrial and Engineering Chemistry* **123**, 509–516 (2023).

5. Grüne, M. & Schweizer, S. Lanthanide-doped glass light guides with bright luminance in the green spectral range. *Opt Mater (Amst)* **142**, 113947 (2023).

6. Saito, S., Hashimoto, R., Hwang, J. & Nunoue, S. InGaN Light-Emitting Diodes on *c* -Face Sapphire Substrates in Green Gap Spectral Range. *Applied Physics Express* **6**, 111004 (2013).

7. Gorrotxategi, P., Consonni, M. & Gasse, A. Optical efficiency characterization of LED phosphors using a double integrating sphere system. *Journal of Solid State Lighting* **2**, 1 (2015).
